# Correction: Chemically-informed active learning enables data-efficient multi-objective optimization of self-healing polyurethanes

**DOI:** 10.1039/d6sc90012g

**Published:** 2026-01-22

**Authors:** Kang Liang, Xinke Qi, Xu Xiao, Li Wang, Jinglai Zhang

**Affiliations:** a Henan Key Laboratory of Protection and Safety Energy Storage of Light Metal Materials, College of Chemistry and Molecular Sciences, Henan University Kaifeng 475004 China chemwangl@henu.edu.cn zhangjinglai@henu.edu.cn

## Abstract

Correction for ‘Chemically-informed active learning enables data-efficient multi-objective optimization of self-healing polyurethanes’ by Kang Liang *et al.*, *Chem. Sci.*, 2026, https://doi.org/10.1039/D5SC07752D.

The authors regret that in the original manuscript, there was a URL included in error in the Acknowledgements section. The Acknowledgements should therefore appear as below.

The Royal Society of Chemistry apologises for these errors and any consequent inconvenience to authors and readers.

